# Metabolic and immunological changes in transition dairy cows: A review

**DOI:** 10.14202/vetworld.2017.1367-1377

**Published:** 2017-11-24

**Authors:** Pratik Ramesh Wankhade, A. Manimaran, A. Kumaresan, S. Jeyakumar, K. P. Ramesha, V. Sejian, D. Rajendran, Minu Rachel Varghese

**Affiliations:** 1 Livestock Research Centre, Southern Regional Station, ICAR-National Dairy Research Institute, Adugodi, Bengaluru - 560 030, Karnataka, India; 2 Southern Regional Station, ICAR-National Dairy Research Institute, Adugodi, Bengaluru - 560 030, Karnataka, India; 3 Division of Animal Physiology, ICAR-National Institute of Animal Nutrition and Physiology, Adugodi, Bengaluru - 560 030, Karnataka, India; 4 Division of Animal Nutrition, ICAR-National Institute of Animal Nutrition and Physiology, Adugodi, Bengaluru - 560 030, Karnataka, India; 5 Dairy Production Section, Southern Regional Station, ICAR-National Dairy Research Institute, Adugodi, Bengaluru - 560 030, Karnataka, India

**Keywords:** acute phase proteins, dairy cows, inflammatory cytokines, negative energy balance, transition period

## Abstract

Smooth transition from pregnancy to lactation is important for high productive and reproductive performance during later postpartum period in dairy animals. On the other hand, the poor transition often leads to huge economic loss to dairy farmers due to compromised production and reproduction. Therefore, understanding the causes and consequence of metabolic changes during the transition period is very important for postpartum health management. In this review, metabolic changes with reference to negative energy balance in transition cow and its effect on health and reproduction during the later postpartum period in dairy animals are discussed besides the role of metabolic inflammation in postpartum performance in dairy animals.

## Introduction

In dairy cattle, the period between 3 weeks before and 3 weeks after parturition is called as transition period, which is one of the most critical physiological stage since most of the metabolic and infectious diseases occur during this period [1,2]. Transition period health is an important determinant of subsequent production and reproductive performance of dairy animals. On the other hand, the occurrence of health problems during the transition period is a major risk factor for subsequent productive and reproductive performance [3]. Higher demand of energy and nutrients for the synthesis of colostrum and milk coupled with decreased feed intake force the transition cows to undergo negative energy balance (NEB) and micronutrient deficiencies. The NEB stimulates cows to mobilize body fat in the form of non-esterified fatty acids (NEFA) and subsequent accumulation of beta-hydroxybutyric acid (BHBA) in the blood. Although these changes are normal adaptive process in high yielding cows, when a cow fails to adapt to this metabolic challenge, several metabolic and infectious disorders occur and affect the productive and reproductive efficiency beyond the transition period.

The poor transition from pregnant to lactation stage often results in the loss of 10-20 lbs (4.54-9.07 kg) of peak milk yield [4], which could equal to 2000-4000 lbs (907.18-1814.37 kg) of untapped milk yield. Despite significant advances in understanding of transition cow biology, higher incidence of metabolic and infectious diseases has been reported during early lactation [5,6]. The incidence of metabolic disorders (such as milk fever, displacement of abomasum, fatty liver syndrome, and ketosis), mammary gland infections (mastitis and udder edema), and reproductive disorders (such as dystokia, retained placenta, and uterine infections) have been reported from 7.8 to 16.8, 2.8 to 12.6, and 6.7 to 19.2%, respectively, in high-producing herds [7,8]. Therefore, a smooth transition is important for minimizing health problems and optimizing productivity and profitability for the forthcoming lactation. Early identification of these diseases may be useful to overcome future production losses [9].

In this review, we discuss about metabolism and inflammation during the transition period with reference to postpartum performance.

## Metabolic Changes during Peripartum Period: Causes and Consequences

**Figure-1:**
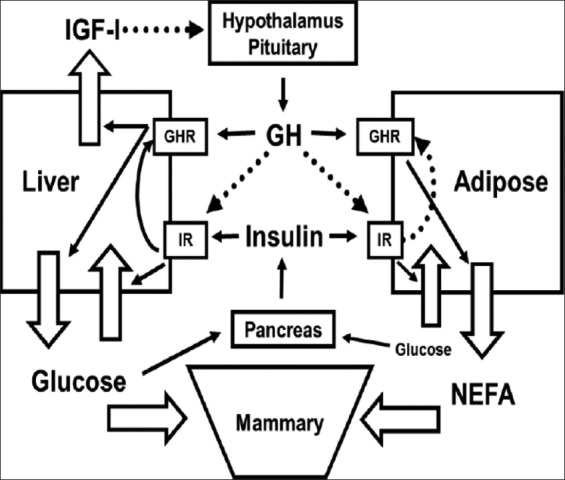
Interaction between growth hormone (GH) and insulin in postpartum dairy cows. Solid lines indicate stimulatory actions, while broken lines infer negative feedback or inhibitory actions. IR: Insulin receptor, GHR: GH receptor [12].

The periparturient period in dairy cows is characterized by profound endocrine and metabolic changes to meet out the milk production during early lactation [10]. Among the various hormonal changes, alteration of growth hormone (GH) axis is well understood [11]. Increased GH concentration during early lactation stimulates hepatic gluconeogenesis to increase glucose supply. Simultaneously, GH also creates an insulin resistance, which prevents the glucose utilization by the liver, muscle, or adipose tissue and stimulates lipolysis, which mobilizes the fatty acids (mainly NEFA) for milk fat synthesis or used as an energy source to some extent in the postpartum cow [1]. Altogether, the gluconeogenesis-mediated more glucose production and lipolysis-mediated fatty acids are directly available for milk synthesis (Figure-1).

Despite these homeorhetic mechanisms, the glucose demand is more during early lactation, particularly in high yielding animals resulting in hypoglycemic state. Although this hypoglycemic state is advantageous in some point of view (e.g., it keeps insulin and insulin-like growth factor (IGF)-1 levels low and thus lesser IGF-1-mediated negative feedback inhibition of GH secretion), inadequate glucose supply leads to the incomplete or partial oxidation of NEFA, which increases the ketone bodies concentration (primarily BHBA) during early postpartum period [13]. This excessive blood NEFA and BHBA has been associated with following unwanted peripartum complications:

- Suppression of dry matter intake (DMI)
- Immunosuppression
- Increased peripartum complications and consequent infertility problems
- Decreased milk production.

## Suppression of Feed or DMI during Transition Period

It is now well established that DMI decreases as calving approaches. About 30% decrease in DMI has been reported during transition period [14,15]. The mechanism for decreased feed intake during transition period was proposed by Allen and Bradford [16], through hepatic oxidation theory. They reported that feeding behavior or feed intake is controlled by feeding center (i.e., brain) through the firing of hepatic vagus nerve depending on adenosine triphosphate (ATP) concentration in the liver. When fuels are oxidized and more are ATPs are produced, there will be less firing on satiety center, while less oxidation-mediated depletion of ATP pool results in more firing or stimulation of feed intake. However, the exact mechanism by which ATP-mediated firing rate of nerve and consequent regulation of feed intake is not thoroughly understood. Among the various fuel sources, fatty acids (dietary or mobilized from body fat), propionate (from microbial fermentation), lactate (produced from glucose in muscle and gut tissues), and amino acids (from protein breakdown) are important fuels in ruminants [16]. Since the fat mobilization in the form of NEFA is the common phenomenon during the transition period, it is likely to suppress the feed intake during this period. However, the magnitude of fat mobilization depends on plasma insulin concentration and its sensitivity to target organs. Higher plasma insulin concentrations and higher sensitivity (lower resistance) stimulate fat synthesis or fat deposition by tissue and *vice versa*. It is reported that plasma insulin concentration and its sensitivity decrease up to 50% or more from several weeks before calving, which consequently increase the plasma NEFA levels.

Besides the feed intake, an appropriate insulin action is important for accurate nutrient partitioning toward the fetus and the mammary gland during the transition period, and an impaired insulin function during the transition period is believed to be associated with elevated body condition and body fat mobilization. Recently, Weber *et al*. [17] demonstrated a minor influence of different degrees of body fat mobilization on insulin metabolism in cows during the transition period, while a significant decrease in the glucose-dependent release of insulin during postpartum period is the most important reason for the impaired insulin action after calving. Bossaert *et al*. [18] found that cows with lower NEFA concentrations during the dry period tended to have an earlier resumption of ovarian activity and decreased plasma insulin response to glucose during the postpartum period. Zarrin *et al*. [19] reported that the effects of hyperketonemia on plasma glucose concentrations are similar before and after calving, but that insulin concentration differed between before and after parturition. Together, it implies that either decreased insulin concentration or its sensitivity is an important risk factor for periparturient complications.

## Immunosuppression in Transition Cows

The bovine innate and acquired immunity are suboptimal during peripartum period, especially during transition period [20]. Although the exact reason for immunosuppression during transition period is not clear, studies with mastectomized cows clearly indicated that gestation and calving or lactation are not the primary factors but the metabolic changes are important determinants of immunosuppression [21]. On the other hand, NEFA and BHBA are major metabolic indicators in large ruminants, and these levels are often increased in blood when animals undergo NEB [22]. Several studies reported that these metabolites are responsible for a suppression of immune responsiveness that consequently leads to the occurrence of metabolic and infectious diseases [20,23-26]. Further, the interdependent nature of the immune and metabolic systems in transition cows were demonstrated through several studies that metabolic diseases (e.g., milk fever and ketosis) put cows at higher risk for infectious diseases (e.g., metritis [Met]) and *vice-versa* [27-29]. Besides, the body condition score (BCS) during the transition period is also an important determinant of immunity in animals.

Scalia *et al*. [30] conducted *in vitro* study to examine the influence of NEFA on bovine polymorphonuclear leukocytes at physiologically relevant concentrations in the blood of cows undergoing high, moderate, or low fat mobilization intensity (2, 1, 0.5, 0.25, 0.125, and 0.0625 mM) and found that the highest concentration of NEFA was associated with a dramatic increase of phagocytosis-associated oxidative burst activities with a reduction in cell viability (48.0 vs. 97.5% in control samples) and marked increase of necrosis (49.4 vs. 0.5%). Hammon *et al*. [31] reported that cows that developed postpartum Met had significantly greater blood NEFA and lower DMI from 2 weeks before calving and greater BHBA during 1-4 weeks after parturition, suggesting that cows experiencing NEB before or around calving are predisposed to periparturient immune suppression. NEB-mediated alterations of gene expression and metabolites (NEFA or BHBA) have important role in uterine immunity. The higher concentrations of NEFA and BHBA during pre-partum were associated with postpartum Met and endometritis in cows, and it could be mediated through impairment of neutrophils function [32]. Giuliodori *et al*. [33] reported that higher pre-partum NEFA and postpartum BHBA are important risk factors for the development of endometritis.

Although most of the transition cows apparently experience inflammation for some period, the magnitude of this inflammatory condition varies greatly among cows. Bertoni *et al*. [25] studied the relationship between liver function, inflammation, milk yield, and fertility in multiparous dairy cows and found that cows in the lower (LO) and intermediate lower (INLO) quartiles of liver activity index (LAI) had more severe inflammations with high concentrations of haptoglobin (0.77 and 0.61 g/L, respectively) during the 1^st^ week of lactation compared with cows in the upper (UP) and intermediate UP quartiles of LAI (haptoglobin: 0.28 and 0.45 g/L, respectively). The INLO group exhibited more days open (139 vs. 93), services per pregnancy (2.68 vs. 1.65), and lower milk yield (38.3 vs. 40.8 kg/day at 28 DIM) compared with the cows in UP group. The cows in LO group had lowest milk yield (34.1 kg/day at 28 DIM). They inferred that cows with lower LAI had a more pronounced inflammatory conditions during the 1^st^ month of lactation, with poor productive and reproductive performance than cows with higher LAI scores.

Inflammation is believed to be an important event during parturition as well as during early postpartum. Occurrences of overt inflammatory reaction without clear signs of infections or any pathological conditions were also reported during transition period [24]. Therefore, the pattern of inflammation during transition period particularly during early lactation is a deciding factor of long-term outcomes. Bradford [34] suggested that brief spikes in inflammatory signals that are resolved in the first 3-4 days of lactation may aid in physiological adaptations to lactation and the end of pregnancy. However, failure to resolve rapidly these inflammatory reactions or signals may lead to an adverse impact on productivity, health, and fertility. In the uterus, cellular defense against bacterial contaminants is mainly provided by leukocytes, in which neutrophils are the primary defense molecule [35]. Although the cytokines are believed to play an important role in neutrophil migration and clearance of pathogens, higher or excessive expressions of pro-inflammatory cytokines are often associated with greater inflammation during the 1^st^ or 2^nd^ week postpartum [36,37], while their lower expression in the endometrium immediately after calving impaired the chemotaxis and activation of neutrophils and led to development of endometritis in cows [38].

## NEFA and BHBA Level as a Predictive Marker of Transition Cow Health and Performance

NEB is an important characteristic of transition cow particularly in high yielding animals, due to the integrative outcome of reduced intake and higher demand for maintenance and production. NEFA and BHBA are important energy metabolites that are traditionally used as indicators of NEB during transition period [39]. An elevation of NEFA and BHBA during the transition period is the metabolic hallmark of the transition from pregnancy to lactation [40] particularly in high-yielding dairy cows. However, an excessive elevation of these metabolites is often associated with poor productive and reproductive performance. The release of NEFA into the blood provides energy to tissues; however, the bovine liver has a limited capacity to metabolize NEFA into triglycerides (TAG). When the limit is reached, the TAG accumulates in the liver, and acetyl CoA (resulting from oxidation of fatty acids) is not utilized in the tricarboxylic acid cycle which is converted into ketone bodies such as acetone, acetoacetate, and BHBA [41] which may appear in the blood, milk, and urine [10]. Excessive accumulation of TAG in the liver impairs its normal function [42]. The synthesis and accumulation of TAG in the liver are related to the concentration of NEFA in the blood; therefore, cows with lipolysis are at high risk to develop fatty liver syndrome [10]. In addition, development of fatty liver has been found to impair the gluconeogenic activity of the liver, which lowers blood glucose and decreases insulin secretion. This, in turn, would support greater lipid mobilization and increased rate of fatty acid uptake by the liver and increased ketogenesis [43]. An imbalance of energy requirement and nutrients intake often leads to various metabolic disorders such as fatty liver, ketosis (clinical or subclinical), ruminal acidosis (subacute or acute), milk fever (subclinical or clinical), and disturbed immune function (retained placenta, Met, and mastitis). The collective effects of all these challenges are leads to reduced fertility and milk production in short and long term. Due to direct and indirect relationship of metabolites with productive and reproductive performance, these molecules have been extensively used for assessment of herd or individual animal health performance. However, the only concerns with these molecules are different threshold level practised across the countries and studies (Table-1) [14,39,44-56]. For instance, threshold concentration of NEFA and BHBA from ≥0.3 to 0.5 mEq/L and ≥0.6 to 0.8 mmol/L, respectively, during prepartum period and from ≥0.7 to 1.0 mEq/L and 1.0 to 1.4 mmol/L, respectively, during postpartum period were used to evaluate the individual cow association with negative outcome in various studies [57]. Although management systems, parity, yield of animals, period of sampling, and targeted disease outcome were the possible explanations for these variations, underlying physiological mechanisms are poorly understood. To establish the cutoff or threshold values of NEFA and BHBA, Ospina *et al*. [48,49,58] conducted a large-scale study in 100 herds (1440 prepartum and 1318 postpartum cows; 35% primiparous and 65% multiparous cows) through sampling of 2758 transition cows (14 to 2 days before calving and 3-14 days after calving) and established critical threshold values for prepartum NEFA and postpartum NEFA and BHBA to predict the disease (displaced abomasum [DA], clinical ketosis [CK], Met, and retained fetal membranes (RFM), within 30 days in milk (DIM), and impact on fertility within 70 days after voluntary waiting period [VWP]) risk ratio. They established threshold level for prepartum NEFA concentration as 0.3 mEq/L and postpartum NEFA concentration as 0.6 mEq/L and 10 mg/dL as postpartum BHBA threshold level. Animals with NEFA and BHBA concentrations above these threshold levels during prepartum and postpartum period had 2 and 4 times higher disease incidence, respectively. They also found that the animals with above critical prepartum NEFA level were 20% less likely become pregnant after 70 days of VWP, while cows with above critical levels of postpartum NEFA and or BHBA were 13-16% less likely to become pregnant and multiparous cows suffered more than primiparous cows. Overall, herd with >15% of transition cows above the critical value of metabolites had slightly greater disease incidence, poor reproductive, and productive performance than herd with <15% cows above critical values. It may be inferred from the above-cited findings that measurement of energy indicators is a useful tool for herd health evaluation and improved transition cow management.

**Table-1:** a. Reported associations between pre- and post-partum NEFA concentrations and postpartum disease risk in dairy animals.

Cut points	Time	Disease	Risk factor/risk ratio	References
<0.5-2 mM	Last week before calving	NS	NS	[14]
0.5-1 mM	Last week before calving	NS	NS	[44]
≥0.37 mmol/l	−14 to−2 days	RFM	2.4	[45]
≥0.38 mmol/l	−14 to−2 days	RFM or Met	2.1	[45]
≥0.29 mmol/l	−14 to−2 days	Any disease	1.6	[45]
≥0.36 mmol/l	+3 to 14 days	RFM or Met	13.2	[45]
≥0.57 mmol/l	+3 to 14 days	Any disease	1.9	[45]
≥0.4 mmol/l	−21 to−3 days	Any disease	4.1	[46]
≥0.5 mmol/l	−21 to−3 days	Any disease	4.2	[46]
≥0.6 mmol/l	−21 to−3 days	Any disease	6.4	[46]
≥0.5 mmol/l	+3 to 21 days	Any disease	2.1	[46]
≥0.6 mmol/l	+3 to 21 days	Any disease	5.0	[46]
≥0.7 mmol/l	+3 to 21 days	Any disease	7.1	[46]
≥0.3 mmol/l	−1 week	RFM	1.6	[47]
0.29 mEq/L	Prepartum	DA, CK, Met and RFM, or any of these three	NS	[48]
0.26 mEq/L	Prepartum	CK	1.8	[48]
0.37 mEq/L	Prepartum	Met, RFM, or both	2.2	[48]
0.57 mEq/L	Postpartum	DA, CK, and Met and RFM, or any of these three	4.4	[48]
0.57 mEq/L	Postpartum	CK	5.0	[48]
0.36 mEq/L	Postpartum	Met, RP, or both	17	[48]
≥0.72 mEq/L	Postpartum	Decreased milk yield	NS	[49]
>0.4 mmol/L	7-10 days before calving	Increased risk of DA, RFM, Culling before 60 days in milk, less milk production in first 4 months of lactation	NS	[50]
0.3 mmol/L	1 week before calving	CE and SCE	9.1 and 12.1	[51]
0.84±0.06 mmol/L	12-24 h after calving	RFM	NS	[52]
≥0.5 mEq/L	1 week before calving	Only in multiparous cows, 3.0±1.5 kg/day per cow milk loss and odds of pregnancy at first AI were lower	NS	[53]
≥1.0 mEq/L	+1 week of calving	Odds of pregnancy at first AI were lower	NS	[53]
>400 mmol/L	Up to postpartum 6 weeks	Ketosis	NS	[54]
0.74±0.29 mmol/L	−5 days	Clinically healthy	NS	[55]
1.08±0.51 mmol/L	5 days	Clinically healthy	NS	[55]
0.76±0.43 mmol/L	10 days	Clinically healthy	NS	[55]
0.35±0.19 mmol/L	30 days	Clinically healthy	NS	[55]
0.15±0.10 mmol/L	60 days	Clinically healthy	NS	[55]

b. Reported associations between pre- and post-partum BHBA concentrations and postpartum disease risk in dairy animals				

≥670 mmol/l	3-14 days	Met	1.5	[45]
≥960 mmol/l	3-14 days	Any disease	3.1	[45]
≥960 mmol/l	3-21 days	Any disease	2.8	[46]
≥1150 mmol/l	3-21 days	Any disease	3.5	[46]
≥1340 mmol/l	3-21 days	Any disease	4.2	[46]
≥1536 mmol/l	3-21 days	Any disease	5.6	[46]
≥1728 mmol/l	3-21 days	Any disease	6.5	[46]
≥1920 mmol/l	3-21 days	Any disease	5.0	[46]
≥1200 mmol/l	1-7 days	Met	3.35	[39]
≥1400 mmol/L	1-2 weeks after calving	Subclinical ketosis	NS	[39]
≥1100 mmol/L	Postpartum period	SCE	NS	[56]
10 mg/dL	Postpartum	DA, CK, Met and RFM, or any of these three	4.4	[48]
10 mg/dL	Postpartum	CK	4.9	[48]
7 mg/dL	Postpartum	Met, RFM, or both	2.3	[48]
10 mg/dL	Postpartum	Decreased milk yield	NS	[49]
>1200-1400 mmol/L	1-2 week after calving	Increased risk of DA, Met, endometritis, ketosis, prolonged uterine anovulation, increased severity of mastitis, lower milk production in early lactation	NS	[50]
0.51±0.08 mmol/L	12-24 hrs after calving	RFM	NS	[52]
≥800 mmol/L	−1 week of calving	Associated with milk loss of 4.4±1.7 kg/day per cow	NS	[53]
0.81±0.27 mmol/L	−5 days	Clinically healthy	NS	[55]
1.29±0.37 mmol/L	5 days	Clinically healthy	NS	[55]
1.03±0.43 mmol/L	10 days	Clinically healthy	NS	[55]
0.80±0.24 mmol/L	30 days	Clinically healthy	NS	[55]
0.66±0.21 mmol/L	60 days	Clinically healthy	NS	[55]
≥670 mmol/l	3-14 days	Met	1.5	[45]

NS=Not specified; days with negative symbol indicates before calving, while days with positive symbol indicate after calving. Met=Metritis, RFM=Retained fetal membranes, DA=Displaced abomasum, CK=Clinical ketosis, CE=Clinical endometritis, SCE=Subclinical endometritis, NEFA=Non-esterified fatty acid, BHBA=Beta-hydroxybutyric acid

## Relationship of Energy Metabolites Level with Health and Reproduction

Huzzey *et al*. [9] reported that multiparous cows with more than one disorder or that died by 30 DIM had greater NEFA concentrations during few weeks before calving, whereas cows with only one disorder after calving had greater NEFA concentration before 1 week only. Primiparous cows with more than one disorder or that died by 30 DIM had greater NEFA concentration during week −2 to −1. Huzzey *et al*. [59] observed that animals with ≥0.30 mEq/L serum NEFA concentration during prepartum period were twice as likely to develop one or more diseases (DA, CK, Met, and RFM). Similarly, animals with postpartum serum NEFA and BHBA concentration ≥about 0.60 mEq/L and 10 mg/dL, respectively, had 4 times higher risk for developing these diseases. Zhang *et al*. [60] found greater serum BHBA concentration in cows with ketosis (1014 ± 140 μmol/L) than control cows (504 ± 140 μmol/L) particularly from 4 weeks before parturition to until 4 weeks after parturition. They also found no interaction between health status and week of blood sample collection on serum NEFA level. Abuajamieh *et al*. [61] found no significant differences in either pre- or post-calving BHBA and NEFA levels between healthy and CK cows although the ketotic cows had increased post-calving circulating NEFA and BHBA concentrations than the healthy cows. Shin *et al*. [54] reported that the higher BCS during pre- and post-partum period and increased NEFA concentration during postpartum period were associated with ketosis, increased reproductive disorders, and decreased reproductive performance in dairy cows.

Now, it is clear that there is a complex interplay between the endocrine systems controlling metabolism, the ovary, and the immune system of the cow. Kumari *et al*. [62] studied the metabolic indicators for RFM in Zebu (Sahiwal) and Karan Fries (KF) crossbred dairy cattle and found that the plasma NEFA concentrations were significantly (p<0.05) higher on the day 14, day 7, day 5, day 3, and day 1 preceding calving and day 0, day 1, and day 2 in KF cows affected with RFM than in cows that expelled the fetal membranes normally. Furthermore, in Zebu cows, the concentrations of NEFA were significantly (p<0.05) higher in RFM-affected cows on the day –5, day –3, day –1, day 0, day 1, and day 2 than those in normal cows. On analyzing BHBA concentration in KF cows affected with RFM, they observed significantly (p<0.05) higher plasma BHBA concentrations on day –7, day –5, day –3, day –1, day 0, day 1, and day 2 than in normal cows. Similarly, in Zebu cows that had RFM, the BHBA concentrations were significantly (p<0.05) higher on the day –5, day –3, day –1, day 0, day 1, and day 2 than in cows that expelled the fetal membranes normally. Moretti *et al*. [63] found that higher concentration of BHBA from 7 to 2 days before and from 48 to 72 h after parturition in RFM cows as compared to healthy control cows. However, they found no difference in NEFA level over time or between groups. Civelek *et al*. [52] also reported that energy imbalance and postpartum NEB may contribute to the development of RFM. Castro *et al*. [64] studied the influence of metabolic energy status during dry period on the postpartum resumption of ovarian activity in dairy cows and found that energy balance (EB) during the dry period was higher in ovulatory (presence of luteal activity within 3-week postpartum) than in anovulatory (without luteal activity within 3 weeks) cows. In contrast, no differences of EB were observed between groups during the postpartum period. The level of BHBA did not differ between groups and ovulatory cows that had higher BCS than an ovulatory cows during the postpartum period. They suggested that EB is closely associated with BCS and the resumption of postpartum ovarian function. Huzzey *et al*. [65] did not found an association between prepartum plasma NEFA concentration and risk of conception. However, they found that an increase in plasma BHBA in the postpartum week was negatively associated with conception. The higher BCS during prepartum and postpartum period and increased NEFA levels during postpartum were associated with ketosis, increased reproductive disorders, and decreased reproductive performance in dairy cows [54].

## Effect of NEB on Milk Production

Although NEB during early lactation is frequently blamed for various metabolic and reproductive disorders [1,66], adverse effects of NEB on milk yield are not clear. During early lactation, the relationship between nutrient supply and milk production appears to be uncoupled where milk yield is increasing toward peak while intake or energy balance is simultaneously decreasing toward its lowest point particularly during the immediate postpartum period (1-10 DIM) and then increases toward positive energy balance. This uncoupling continues for at least 6-8 weeks. McGuire *et al*. [67] reported that cows were back to positive EB by 4 weeks after calving. Moallem *et al*. [68] also reported that positive EB by 4-5 weeks after calving, while other researcher reported longer duration of 6-8 weeks to return positive EB [42]. Although fat mobilization is important reason for compensation of energy requirement during early lactation, the same phenomenon does not continue during mid or later stage of lactation [69], where milk yield itself is the main determining factor of feed intake [70]. In other words, whenever, animal is in NEB, additional metabolizable energy (ME) would increase the milk production, while under positive energy balance, providing additional ME will improve efficiency without increasing of milk yield [69]. These findings suggest that milk synthesis is the single most factor deciding the energy balance of individual cow.

Moyes *et al*. [71] indicated that increased NEFA and BHBA concentrations are related to the development of mastitis. Huzzey *et al*. [65] reported that for each unit increase (1 mEq/L) in NEFA concentration during prepartum period (week - 2) caused 1465 kg decrease in average milk yield. The increase in NEFA concentration during postpartum period increased milk production in primiparous cows and decreased in multiparous cows. Ospina *et al*. [48,49] studied the associations of energy indicators with subsequent milk production (assessed as mature-equivalent 305-day lactation milk, predicted at approximately 120 DIM). Regardless of parity, animals with >0.3 mEq/L of NEFA during the prepartum period had nearly 700 kg less projected milk than animals with lower concentrations. During the postpartum period, they found differential associations of energy-related analytes with milk production depending on the parity. In primiparous cows (heifers), postpartum NEFA concentrations ≥0.6 mEq/L and BHBA concentrations ≥9 mg/dL were associated with increased milk yield. In multiparous cows, postpartum NEFA concentrations ≥0.7 mEq/L and BHBA concentrations ≥10 mg/dL were associated with lower predicted milk yield. Zhang *et al*. [60] reported that average daily milk production in ketotic cows (35.25±2.53 kg/day) was lower than healthy cows (42.16±2.53 kg/day) particularly in the week when the disease was diagnosed (27.02±2.02 vs. 40.31±1.62 kg/day, respectively). Abuajamieh *et al*. [61] studied the inflammatory biomarkers associated with ketosis in periparturient Holstein cows of two different farms and found that the ketotic cows had reduced milk production as compared to healthy cows (28.3 vs. 38.6 kg/day; in farm 1 and 32.8 vs. 36.0 kg/day in farm 2). They also found no differences in fat-corrected milk, somatic cell count, and calculated energy balance between both groups.

## Metabolic Inflammation: A Deciding Factor of Postpartum Performance

**Figure-2:**
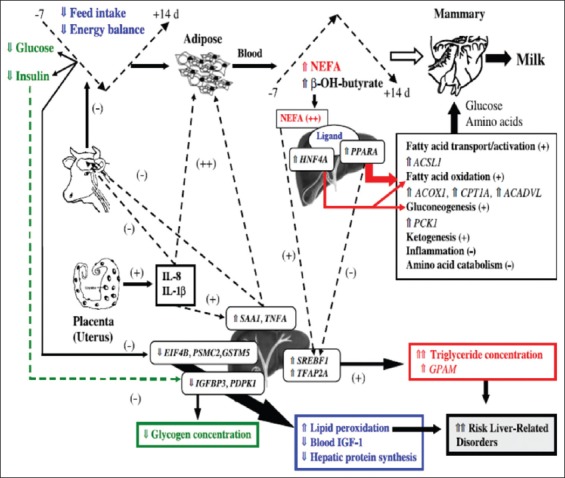
Suggested mechanism for liver-related periparturient metabolic disorders in dairy cows [72].

Among the tissues that support milk production, such as brain, endocrine glands, digestive tract, adipose tissue, skeletal muscle, and immune systems, the liver is pivotal organ because it coordinates nutrient metabolism with the endocrinology of the cow. Loor *et al*. [72] conducted the first experiment to understand the relationship between bovine liver transcriptome (through liver biopsy using a microarray technique) and serum metabolic indicators (NEFA, total protein, urea nitrogen, BHBA, glucose, and insulin), liver composition (total lipid, triacylglycerol, and glycogen), body weight, BCS, and energy balance during periparturient period (−65, −30, −14, +1, +14, +28, and +49 days relative to parturition) and explained the physiological events in placenta, adipose, and liver that leads to liver-related health disorders in dairy cows (Figure-2). They observed positive associations between serum NEFA and SAA mRNA expression while transcript levels of acyl-CoA synthetase long-chain family member 1, peroxisome proliferator activated receptor-α (PPARA), and transcription factor AP-2 alpha 6 were positively correlated with serum BHBA. They also revealed based on certain gene expression that metabolic adaptations start well ahead of parturition and it is mainly regulated by hepatic inflammatory responses during periparturient period which initiate or augment adipose catabolism rather than by hormonal environment. It clearly shows that cytokines, acute-phase proteins, and serum NEFA are key players in periparturient cow metabolism.

Loor *et al*. [72] proposed mechanism for liver-related metabolic disorder that interleukin-8 (IL-8) and IL-1β secreted from placenta directly upregulate the expression of SAA1 and tumor necrosis factor-α (TNF-α) in the liver. These cytokines and APP have negative effects on satiety center of the brain which leads to hypoinsulinemia, hypoglycemia, and depressed feed intake results in NEB and consequent increase of adipose tissue lipolysis. Immune molecules from placenta and/or liver may further stimulate lipolysis and markedly increase the circulatory NEFA and BHBA levels. Circulating NEFA is endogenous ligands for PPARA and hepatocyte nuclear factor-4A resulting in upregulation of net hepatic glucose synthesis and thereby sparing the glucose and amino acids for milk synthesis through upregulation and activation of genes related to fatty acid oxidation, ketogenesis, and gluconeogenesis.

Further, cytokines and NEFA-mediated activation of sterol regulatory element binding transcription factor 1 cause greater concentrations of liver triacylglycerol. Hypoinsulinemia and limited amino acids (leucine, isoleucine, and valine) cause downregulation of insulin-like growth factor binding protein 3, eukaryotic translation initiation factor 4B, 3-phosphoinositide-dependent protein kinase-1, and/or proteasome (prosome, macropain) 26S subunit, ATPase 2 resulting in decreased hepatic protein synthesis, circulating blood IGF-I, and liver glycogen. Downregulation of antioxidant, glutathione S-transferase M5, expression may increase lipid peroxidation in the liver. Collectively, both reduced the capacity to detoxify free radicals and greater triacylglycerol accumulation in liver increase the risk for hepatic periparturient health disorders (Figure-2).

Loor *et al*. [73] also reported that copious milk production by the postpartum cow starts the utilization of the carbohydrate resources. Besides, management and/or disease incidence reduce the feed intake and thus enhanced adipose tissue lipolysis and release of NEFA (e.g., palmitic acid and oleic acid) to the liver. Alongside, macrophages in adipose tissue secrete cytokines and other pro-inflammatory mediators into the liver which leads to metabolic inflammation through upregulation of IL-6. The excessive NEFA, cytokine, and BHBA accumulation in liver reduce energy production and metabolism, perhaps due to decreased milk production, but maintain fatty acid oxidation through upregulation of PPAR signaling pathways. Reduced energy production also ensures lesser reactive oxygen species generation in the liver. The IL-6 plays a central role in a wide range of liver-specific functions in cattle such as lipoprotein metabolism, fatty acid oxidation, urea cycle, oxidative stress, transcription regulation, and protein degradation through proteasomes.

Inflammatory cytokines are believed to be a central integrator of metabolism and immune function [74]. Trevisi *et al*. [75] reported that administration of interferon alpha (IFN-α) during transition period resulted in a decrease of BCS along with decreased milk yield, increased haptoglobin and ceruloplasmin, and a slower increase of negative acute phase proteins (albumin, cholesterol, paraoxonase, and Vitamin A) after calving. IFN-α-treated animals also showed a larger decrease of plasma glucose and higher levels of NEFA, BHBA, and reactive oxygen metabolites. They also found IL-6 and TNF-α response before calving with a quick decrease thereafter. All these findings indicate a sustained inflammatory reaction along with metabolic changes in low-dose IFN-α-treated dairy cows. Cows administered with recombinant bovine TNF-α during late pregnancy showed increased hepatic triglycerides (fatty liver) and increased the transcript abundance of metabolic genes related to free fatty acids uptake and storage, while decreased the genes transcript related to gluconeogenesis, suggesting that inflammatory pathways may also be responsible for decreased glucose production in cows with fatty liver [76].

## Conclusion

NEB is an important characteristic of transition cows, and it is a normal adaptive mechanism in high yielding dairy animals. Although changes in the energy and nutrient intake along with hormonal environment toward parturition were thought as major determinants of NEB, recent evidence indicate that hepatic inflammatory responses during periparturient period are an important driving force for fat mobilization. Physiological adaptation to metabolic processes in periparturient cow starts well in advance of parturition, and it is mainly regulated by cytokines, acute-phase proteins, and energy metabolites. Despite a clear understanding of cause and consequence of many periparturient complications, cellular and molecular studies to associate these events are less. At present, several association studies between energy balance during transition period and subsequent postpartum performance are available; however, further studies are required, particularly in moderate yielding Indian dairy cattle, for effective postpartum management.

## Authors’ Contributions

PRW, AM, AK, SJ, KPR, VS, and DR conceptualized the concept of this review paper. PRW, AM, AK and SJ prepared the manuscript. MRV assisted in collecting and compiling the resource material. All authors read and approved the final manuscript.
